# Case-control geographic clustering for residential histories accounting for risk factors and covariates

**DOI:** 10.1186/1476-072X-5-32

**Published:** 2006-08-03

**Authors:** Geoffrey M Jacquez, Jaymie R Meliker, Gillian A AvRuskin, Pierre Goovaerts, Andy Kaufmann, Mark L Wilson, Jerome Nriagu

**Affiliations:** 1 BioMedware, 516 North State Street, Ann Arbor, MI 48104-1236, USA; 2 The University of Michigan, School of Public Health, Ann Arbor, MI, USA

## Abstract

**Background:**

Methods for analyzing space-time variation in risk in case-control studies typically ignore residential mobility. We develop an approach for analyzing case-control data for mobile individuals and apply it to study bladder cancer in 11 counties in southeastern Michigan. At this time data collection is incomplete and no inferences should be drawn – we analyze these data to demonstrate the novel methods. Global, local and focused clustering of residential histories for 219 cases and 437 controls is quantified using time-dependent nearest neighbor relationships. Business address histories for 268 industries that release known or suspected bladder cancer carcinogens are analyzed. A logistic model accounting for smoking, gender, age, race and education specifies the probability of being a case, and is incorporated into the cluster randomization procedures. Sensitivity of clustering to definition of the proximity metric is assessed for 1 to 75 *k *nearest neighbors.

**Results:**

Global clustering is partly explained by the covariates but remains statistically significant at 12 of the 14 levels of *k *considered. After accounting for the covariates 26 Local clusters are found in Lapeer, Ingham, Oakland and Jackson counties, with the clusters in Ingham and Oakland counties appearing in 1950 and persisting to the present. Statistically significant focused clusters are found about the business address histories of 22 industries located in Oakland (19 clusters), Ingham (2) and Jackson (1) counties. Clusters in central and southeastern Oakland County appear in the 1930's and persist to the present day.

**Conclusion:**

These methods provide a systematic approach for evaluating a series of increasingly realistic alternative hypotheses regarding the sources of excess risk. So long as selection of cases and controls is population-based and not geographically biased, these tools can provide insights into geographic risk factors that were not specifically assessed in the case-control study design.

## Background

Pattern recognition plays an important role in the analysis of geographic distributions of human disease, providing an objective basis for evaluating whether pattern on a map may be explained by chance [1]. Only after such an objective evaluation (e.g. finding a statistically significant cluster) is one justified in formulating an explanatory hypothesis or implementing an action to control disease or ameliorate its impact [2]. Dozens of approaches for quantifying pattern on disease maps have been proposed, but many of these are founded on simplistic assumptions such as immobile individuals and that the latency between causative exposures and health events (e.g. diagnosis, death) is negligible [3]. While some methods may account in an appropriate fashion for one or more of these assumptions, to our knowledge none of the presently available methods for geographic clustering of case-control data successfully accounts for all of them. This paper presents a novel approach for evaluating clustering in case-control data that accounts for residential mobility, known risk factors, and covariates. We begin by identifying unrealistic assumptions implicit in commonly used cluster tests, and then describe ways of relaxing these assumptions. We then summarize a recently defined family of statistics (called Q-statistics, [4]) for analyzing clustering in case-control data using residential histories, and introduce extensions that account for known risk factors and covariates. We then apply this new approach to data from an ongoing-study of bladder cancer in 11 counties in southeastern Michigan.

### Limitations of common assumptions of disease clustering

That risk of disease may vary from one geographic sub-population to another, and is time-dependent, is a fact for both infectious and chronic diseases. But most geographic clustering methods employ a static world-view in which individuals are considered immobile, migration between populations does not occur, and in which background disease risks under the null hypothesis are assumed to be time-invariant and uniform through geographic space. In many instances these assumptions are incorrect, and improved approaches founded on more realistic assumptions are needed.

The lack of an appropriate representation of the time dimension is referred to as a "static world-view" [5]. One of the consequences of a static world-view is a failure to adequately represent human mobility. Especially for chronic diseases, causative exposures may occur in the past, and the disease may be manifested only after a lengthy latency period. During this latency period individuals may move from one place of residence to another. This can make it difficult to detect clustering of cases in relation to the spatial distribution of their causative exposures. To date most techniques for analyzing disease patterns have largely ignored human mobility, relying instead on static spatial point distributions to describe place of residence at time of diagnosis or death. Examples include Turnbull's test [6-8], Cuzick and Edward's test [9], Besag and Newell's test [10], the Bernoulli form of the scan test [11,12], Tango's test [13] and a host of others. Recent studies have demonstrated that results based on static spatial point distributions depend critically on the times chosen to observe the system [4]. Especially for chronic diseases with long latencies, human mobility must be accounted for, and techniques based on static point distributions may be inappropriate.

Even after mobility is taken into account excess risk may be due to an aggregation of individuals with high-risk attributes and covariates, such as cigarette smoking and old age. Clustering methods thus must account for individual-level risk factors and covariates, as well as residential mobility. To our knowledge there currently exist no techniques for modeling disease clusters that simultaneously account for human mobility, covariates and known risk factors. In this article we will address each of these needs within the framework of inferential clustering methods.

### Neutral models to account for risk factors and covariates

Before considering techniques for handling human mobility let us consider approaches for identifying clustering of cases above and beyond the clustering expected given the geographic distributions of known risk factors (e.g., smoking) and covariates (such as age, education, socio-economic status, etc). Goovaerts and Jacquez [14] proposed neutral models that relax assumptions of geographically uniform risk and spatial independence under the null hypothesis, and demonstrated the approach for the local Moran's *I *statistic. In this paper we extend the concept to tests for local and focused clustering in case-control data. The idea is to incorporate each individual's probability of being a case based on his/her known risk factors and covariates. We then use this probability to accomplish the assignment of case-control identifiers. The resulting null hypothesis then accounts for the geographic distribution of the covariates and known risk factors. Any observed case clustering thus cannot be attributed to the risk factors and covariates, and instead may be attributable to some other, perhaps unknown, risk factor. Our implementation of this approach is detailed in the methods section.

### The modeling of human mobility

Thorsten Hagerstand [15] proposed constructs for representing the space-time paths formed as individuals move throughout their days that have come to be known as geospatial lifelines [16]. Miller [17] developed approaches for modeling uncertainty in how a person's location changes through time. These techniques are just beginning to be used in the analysis of human health data, as now summarized.

Sinha and Mark [16] employed a Minkowski metric to quantify the dissimilarity between the geospatial lifelines of cases and controls, and suggested their technique could be used to evaluate differences in exposure histories between the case and control populations. The Minkowski metric provides a global measure of dissimilarity between cases and controls; however, it does not facilitate the identification of where or when these dissimilarities occur. Using *k*-function analysis, Han and Rogerson [18,19] evaluated clustering of breast cancer in two New York state counties and detected significant spatial clustering at the global level. Their approach incorporated knowledge of residential locations for both cases and controls, since they analyzed place of residence at specific time slices in the participants life, namely at birth, menarche, and at woman's first birth. The underlying representation is a static spatial point distribution, and the *k*-function analysis does not account for underlying temporal changes in place of residence. In a study of breast cancer incidence on Cape Cod, Ozonoff and colleagues [20,21] assessed case clustering using three different clustering methods and three different latency assumptions. Static spatial point distributions were analyzed using historical place of residence defined by the different latencies. In an earlier case-control study using the Cape Cod data, Paulu et al [22] explored associations between residential location and breast cancer incidence adjusting for individual risk factors. However, their methods analyzed static spatial point distributions that did not fully account for human mobility.

Jacquez et al [4] developed global, local and focused versions of Q-statistics for evaluating clustering in residential histories using case-control data. Their approach is based on a space-time representation that is consistent with Hagerstrand's space-time paths, and that relaxes the assumption of a static world-view. Q-statistics use the residential histories of the participants to evaluate local, global, and focused clustering over a case's life-course relative to the residential histories of the controls. One of the benefits of Q-statistics is their ability to document pattern at spatial and temporal scales that are of direct relevance to individuals, while also providing global statistics for evaluating clustering at the population level. But Jacquez et al [4] did not account for known risk factors and covariates, a need addressed in this paper.

### Inference framework

The techniques detailed in this article have two principal advantages. First, they provide an assessment of clustering that is founded on a realistic representation of residential histories. Second, they use realistic null hypotheses based on known risk factors and covariates. This provides a mechanism for systematically evaluating a set of alternative hypotheses that might plausibly explain the observed clustering, and allows us to rigorously identify those localities and sub-populations with unexplained excess risk.

To illustrate, consider the method of Strong Inference proposed by Platt [23], and which is a modification of Popper's scientific method [24]. Platt suggested that a set of alternative hypotheses be formulated comprising the reasonable explanations for the problem being considered, based on the available data and the researcher's knowledge at that time. As the study advances this set might be expanded as insights are gained. Next, one designs a series of experiments to systematically evaluate each of the alternative hypotheses. These experiments are conducted and the corresponding alternative hypotheses are rejected, leaving the researcher with the one hypothesis that explains the phenomenon under observation. This approach is analogous to that followed by Sir Arthur Conan Doyle's fictitious crime fighter, Sherlock Holmes, who observed, in "The Adventure of the Blanched Soldier"

'*When you have eliminated all which is impossible, then whatever remains, however improbable, must be the truth*."

### Alternative hypotheses

For the present study, we investigate spatial and temporal clustering in bladder cancer cases in southeastern Michigan. After accounting for established risk factors, many cases of bladder cancer remain unexplained [25], and novel techniques such as Q-statistics are needed to shed light on this public health enigma. We proceed by enumerating a set of alternative hypotheses, not necessarily exclusive, that might explain spatial and temporal clustering of bladder cancer. These hypotheses are:

A0: There is global clustering of bladder cancer cases in southeastern Michigan

A1: There is local clustering of bladder cancer cases in southeastern Michigan

A2: The clusters may be explained by known risk factors and covariates

A3: There is focused clustering of bladder cancer cases about industries in excess of that explained by known risk factors and covariates

We then conduct a series of statistical experiments to evaluate each of these alternatives. We reasoned that if clustering persists after accounting for known risk factors and covariates, then it may be attributable to a risk factor not quantified in the original study design.

## Results

The results are summarized in Table 1, and are described below.

**Table 1:** Results of global, local and focused analyses for 14 *k* nearest neighbors.

*k*	*Q*_k_	p(*Q*_k_|ind)	p(*Q*_k_|cov)	$Q_{k}^{F}$	p($Q_{k}^{F}$|ind)	p($Q_{k}^{F}$|cov)
1	0.174901	0.005	0.017	0.127530	0.029	0.068
2	0.349723	0.003	0.005	0.184488	0.041	0.136
3	0.517915	0.002	0.008	0.245975	0.035	0.075
4	0.684462	0.001	0.005	0.309150	0.020	0.070
5	0.855060	0.001	0.005	0.373301	0.012	0.059
6	1.026782	0.001	0.004	0.435352	0.014	0.037
7	1.198437	0.001	0.003	0.497214	0.015	0.035
8	1.369669	0.001	0.004	0.559708	0.008	0.034
9	1.538379	0.001	0.003	0.621404	0.007	0.039
10	1.698601	0.001	0.004	0.678253	0.006	0.044
15	2.515135	0.001	0.016	0.963308	0.021	0.063
25	4.094881	0.003	0.055	1.545931	0.015	0.049
50	8.129378	0.002	0.054	2.975514	0.028	0.067
75	12.149053	0.004	0.047	4.463786	0.012	0.034

Column 1 is the number of nearest neighbors considered (k = 1,...,10, 15, 25, 50, 75); Qk is the value of the global statistic for evaluating clustering of residential histories of cases over the entire study period and study area; p(Qk|ind) is the probability of Qk under the null hypothesis of independence that assumes the cases and controls have equal probability of being a case; p(Qk|cov) is the probability of Qk accounting for smoking, age, gender, education and race; QFk is the focused statistic for assessing clustering about the business address histories of the 268 industries, and is evaluated for all industries simultaneously; p(QFk |ind) is the probability of QFk under the null hypothesis of independence; p(QFk|cov) is the probability of QFk accounting for smoking, age, gender, education and race.

### A0: There is global clustering of bladder cancer cases in southeastern Michigan

We first employed the global test Q_k _to quantify case-control clustering in the residential histories without accounting for known risk factors and covariates. This statistic is large when clustering of many of the residential histories of the cases persists through time. We used the duration-weighted version of the statistic and obtained Global Q_k _values that ranged from 0.0175 at *k *= 1 to 12.149 when 75 nearest neighbors are considered. Using 999 randomization runs we obtained p-values from a minimum of 0.001 to a maximum of 0.005, and all of the 14 levels of *k *nearest neighbors considered were statistically significant (column "p(Q_k_|ind)" in Table 1). We accept hypothesis A0 and conclude there is statistically significant global clustering of the residential histories of bladder cancer cases when smoking and the four covariates are not accounted for.

### A1: There is local clustering of bladder cancer cases in southeastern Michigan

The analysis for A0 did not identify where and when the clusters occur. To identify local case clusters we used the local statistic Q_i,k_that is sensitive to clustering of the residential history of cases about individual cases (a local test through time). This results, for each level of *k*, in an animation showing how the spatial distribution of statistically significant case clusters changes through time. We found persistent case clusters in Oakland, Ingham and Jackson counties. We accept hypothesis A1 and conclude there is persistent case clustering in these three areas of Michigan. But we do not yet know whether these clusters may be explained by smoking and the covariates age, gender, race and education.

### A2: The clusters may be explained by known risk factors and covariates

To account for known risk factors and covariates we used logistic regression to predict the probability of being a case (Equation 8b) as:

$\hat{p}(c_{i}=1|x_{i})=\frac{e^{\left(2.0359-0.0125*Age_{i}-0.9396*Gender_{i}+0.1900*Educate_{i}+0.0557*Race_{i}-0.2438*Cignum_{i}\right)}}{1+e^{\left(2.0359-0.0125*Age_{i}-0.9396*Gender_{i}+0.1900*Educate_{i}+0.0557*Race_{i}-0.2438*Cignum_{i}\right)}}\;\left(\text{Equation }1\right)$

**Figure 1:**
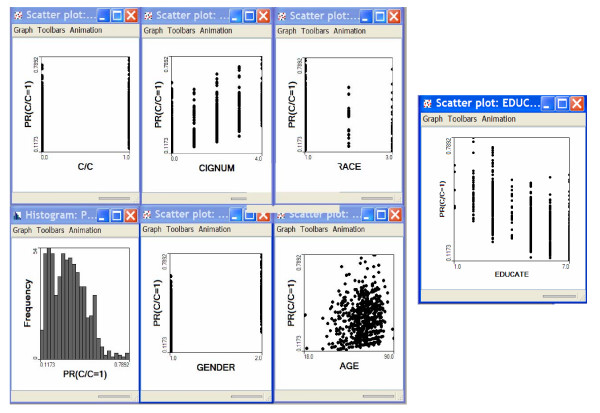
Results from logistic model. "PR(C/C = 1)" is the probability of an individual being a case given the logistic model and the vector of risk factors and covariates for that individual. "C/C" indicates the case control identifier, 0 indicates a control and 1 indicates a case. "CIGNUM" is the number of cigarettes smoked: 0 = never smoked, 1 = smoked < 10 cigarettes daily, 2 = smoked 11–20 cigarettes daily, 3 = smoked 21–30 cigarettes daily, 4 = smoked > 30 cigarettes daily. "RACE" is 1 = white, 2 =black, 3 = other. "EDUCATE" is a participant's level of education attained, 1 = <8 years, 2 = 8–11 years, 3 = 12 years or high school graduate, 4 = post high school training, 5 = some college, 6 = college graduate, 7 = postgraduate education. "GENDER" is 1 = Male, 2 = Female. "AGE" is the participant's age at time of interview.

Not surprisingly, increased smoking is associated with higher probability of being a case; this risk increases with age, and appears elevated for whites and females (Figure 1). Bladder cancer typically afflicts older white males to a greater extent than the remainder of the population [25]; however, in our study, females experience a higher risk because controls are in the process of being frequency matched to cases, and in our dataset more females are cases than controls. Also as expected, being white and completing fewer years of education are associated with a higher probability of being a case.

**Figure 2:**
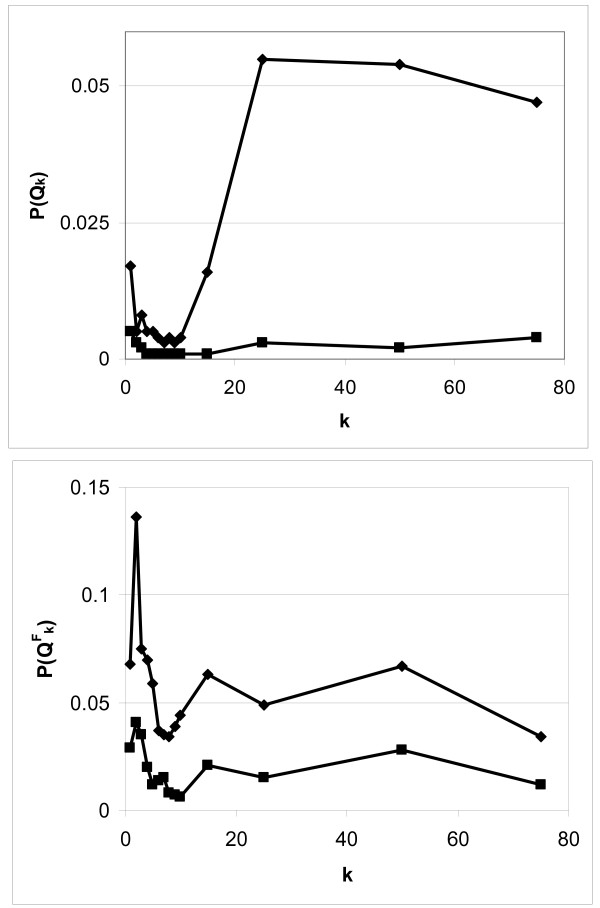
Sensitivity to *k*, the number of nearest neighbors. Global (top graph) and focused (bottom graph) statistics before (diamonds) and after (squares) covariate adjustement. After covariate adjustment the p-value reaches a minimum at *k *= 7 for the global statistic and *k *= 8 for the focused statistic.

We incorporated the probabilities from Equation 1 into the randomization procedure as described in the section "Randomization accounting for risk factors and covariates". We then recalculated the probabilities of both the global and local Q statistics. Because the geometry of the residential histories doesn't change we obtained the same values for the test statistics. For example, Q_k _= 0.855060 at *k *= 5 for both the not- and covariate-adjusted versions. After accounting for risk factors and covariates in the randomization procedure we obtained different p-values. For example, at *k *= 5 the probability before adjustment was 0.001, and after adjustment was 0.005. After covariate adjustment the p-values all increased from 2 to 10 times for all of the levels of *k*. 12 of the 14 levels of *k *considered were still statistically significant at the 0.05 level after covariate adjustment (Figure 2, top). We therefore fail to accept hypothesis A2, and conclude the observed global clustering of residential histories of the cases cannot be explained by smoking, age, gender, race and education.

**Figure 3:**
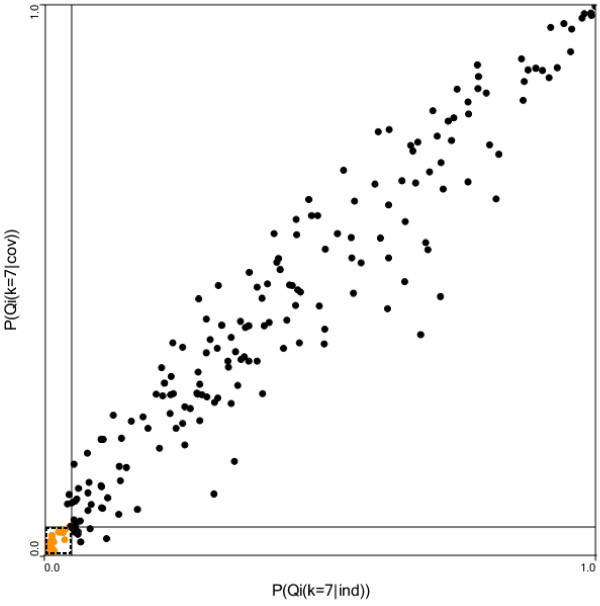
Probability of the local Q statistic at *k *= 7 not accounting for smoking, age, gender, race and education (*x *axis) versus the probability of the local statistic accounting for smoking and these covariates (*y *axis). The 20 points in the lower left quadrant are centers of significant case clusters even after smoking and the four covariates are accounted for. The 6 points in the lower right quadrant were cases that have become significant after covariate adjustment. 4 cases were significant before covariate adjustment but not afterwards.

Figure 3 plots the probability of the local Q statistic under the logistic equation (*y*-axis) versus the probability of the local Q statistic not adjusted for smoking and the four covariates (*x*-axis) at *k *= 7. We use *k *= 7 since this is the number of nearest neighbors for which the global statistic obtained a minimum p-value after covariate adjustment (Figure 2). This graph is divided into four quadrants formed by drawing lines on each axis at p = 0.05. Each point on this graph corresponds to a cluster of *k *= 7 cases whose center is defined by the residential history of the case that is at the center of the cluster. Points in the lower left quadrant defined by p-values less than 0.05 indicate cases that are statistically significant cluster centers even after accounting for smoking and covariates (20 cases). Points in the lower right quadrant are cases that become significant after covariate adjustment (6 cases). Points in the upper left quadrant were significant before covariate adjustment, but not after (4).

Where are these 26 clusters, and do they persist through time? They are found in Lapeer, Ingham, Oakland and Jackson counties (See additional file 1, animation of local clusters after adjustment for covariates). The clusters in Lapeer and Jackson counties are comprised of 1–3 cluster centers, and are ephemeral. The clusters in northwestern Ingham County appear in 1950, concentrate to the northwest of Lansing and persist into 2000. Numerous clusters appear in central and southeastern Oakland County beginning in the 1950's and persist to the present day. We conclude there is statistically significant local clustering after covariate adjustment. This, along with the persistence through time of concentrations of clusters in Ingham and Oakland counties suggests the possible action of a risk factor or covariate yet to be accounted for.

### A3: There is focused clustering of bladder cancer cases about industries in excess of that explained by known risk factors and covariates

Bladder cancers have a multiplicity of possible causative exposures. We constructed a database of 268 industries using the Toxics Release Inventory [26] and the Directory of Michigan Manufacturers. Industries were selected that emit known or suspected bladder cancer carcinogens. We then analyzed clustering about these industries while accounting for smoking and the four covariates. We used the focused statistic $Q_{k}^{F}$ that considers all of the foci simultaneously (a global test) and *Q*_F,k _that evaluates clustering about the F^th ^industry (a local test). We employed the randomization procedures based on the logistic regression. Any focused clusters we find then indicate excess risk beyond that explained by smoking, age, gender, race and education.

We first analyzed the data not adjusted for smoking and the four covariates (Table 1, columns "$Q_{k}^{F}$" and "p($Q_{k}^{F}$|ind). For example, at *k *= 5 we obtained $Q_{k}^{F}$ = 0.309150, with a probability of p($Q_{k}^{F}$|ind) = 0.020. When we adjusted for covariates this p value increased to 0.070 (Table 1, column "p($Q_{k}^{F}$|cov)"). Of the 14 levels of *k *evaluated, all were significant at the 0.05 level before covariate adjustment, 7 were significant after covariate adjustment, and the p-value after covariate adjustment achieved a minimum of 0.034 at *k *= 8 (Figure 2, bottom). After covariate adjustment statistically significant focused clusters are found about the business address histories of 22 industries located in Oakland (19 clusters), Ingham (2) and Jackson (1) counties. Clusters in central and southeastern Oakland County appear in the 1930's and persist to the present day. Approaches for interpreting multiple runs of nearest neighbor analyses have challenged spatial analysts for some time [9,27] and will be a topic of the Discussion section.

Are the 22 industries that have a significant excess of cases in their immediate vicinity grouped in one or more areas of the map, and does this pattern change through time? To answer this question we created a time animation of the business address histories, identifying those industries that were statistically significant focused clusters (See Additional file 2, animation of focused clusters after covariate adjustment, *k *= 8).

It is interesting to note the clustering of 15 statistically significant industries in the southeastern portion of Oakland County. These industries include manufacture of plastics and synthetic resins, perfumes, printing ink, finished rubber and leather products, and industrial organic chemicals. Other industries that produced perfumes, printing ink, finished rubber products, and industrial organic compounds were identified in other parts of the study area, suggesting that these industries may not be responsible for the clusters. On the other hand, one of the industries in the Oakland County cluster was the only manufacturer of finished leather products in the study area from the 1940s–1990s. The prospect of environmental pollution originating from these facilities being associated with bladder cancer is intriguing; however, caution is necessary until the study is complete. We are in the process of obtaining occupational histories to incorporate as risk factors in the logistic regression model, thus creating a neutral model that includes smoking and occupational exposures, along with key covariates. Until then, we cannot rule out occupational exposures in explaining the focused clustering around certain industries. This will be explored in greater detail when participant recruitment into the study and data collection is complete.

## Discussion

We must emphasize that the study from which the data originated is approximately 1/2 way through the data collection phase. We thus cannot draw any inferences from the analysis of these data, and have used them only for example purposes. Once the data collection is complete we intend to rigorously revisit these analyses using the full data set.

We must recognize that 268 industries were considered, and that 14 levels of *k *were analyzed. The minimum p value of the global Q_f _was obtained at *k *= 8 and was 0.034, and the global Q_f _statistic accounts for the number of industries considered. Given an alpha level of 0.05, and the 14 repeated analyses, we would expect 0.7 of these Q_f _to be statistically significant if the null hypothesis were true. We found significant focused clustering 7 of 14 times. It thus appears highly unlikely that the observed global clustering is consistent with the null hypothesis. We thus appear to be justified in inspecting the 268 industries to identify those that are likely to be cluster foci. In the interest of public health it is worth exploring those facilities with the most extreme p-values to single out those that consistently are at the center of a cluster of cases. Once identified, additional epidemiological investigation may be warranted to uncover a biologically plausible exposure, and to determine whether individuals in the vicinity of the operation actually demonstrate a body burden for the suspected carcinogen.

Recent research [16,18,20,22] has sought to address clustering over the life course and during those episodes in life thought to be associated with excess risk (e.g. age at menarche for breast cancer). The methods employed by these studies rely on "snap shot" approaches that employ static spatial point distributions. They attempt to take residential mobility into account by analyzing clustering in residential locations at different points in time, but this approach ignores the residential history formed by connecting the string of locations at which an individual has lived through their lifetime. By modeling residential histories as a series of connected locations that changes through time, we are able to track time spent at different residences, as well as the changing space-time geometry of the residential histories of the study population. We then can incorporate knowledge of both individual- and population-level residential histories into the cluster statistics. This is a significant methodological advance that makes possible handling of the hysteresis – dependency of current state on those that came before – that is the hallmark of disease processes. This is absolutely essential when we seek to address questions regarding changing risk over an individual's life course.

## Conclusion

When considering diseases with long latency such as cancer representation of residential mobility is required whenever risk is associated with place of residence. In these instances, methods such as the Q-statistics are preferred. The added value of the approach demonstrated in this paper is the ability to (1) identify specific individuals whose cancer is not adequately explained by the known risk factors and covariates, and to (2) identify specific industries and facilities that plausibly might explain local excesses of cases not attributable to known risk factors and covariates.

The case-control epidemiological study design provides a wealth of information at the individual level regarding exposures, risks, risk modifiers and covariates. When designing such a study the researcher often is concerned with assessing a few putative exposures, and in determining whether there are significant differences in these exposures between the case and control populations. As such, the case-control design is not inherently spatial, nor is it particularly well suited or even capable of assessing risk factors other than those specified in the original design.

The approach described in this paper may prove to be a highly useful addition to the traditional aspatial case-control design because it allows researchers to identify local groups of individuals whose risk exceeds that accounted for by the known risk factors and covariates incorporated under the designed study. Further, the ability via the local and focused tests to quantify pockets of cases whose excess risk might be attributable to specific locations or point sources is a powerful addition to the inferential toolbox. While such a tool can never of itself assess the dose-response relationship necessary to attribute risk to a specific location or point source, the ability to temporally and geographically localize the putative exposure source makes it possible to begin the assessment of dose-response relationships. Once such a putative focus has been identified, the next step may involve techniques for modeling exposure that will provide a more accurate and detailed description of the spatial and temporal variability in exposure. And once a specific point source is identified, the task of quantifying the type and quantity of releases of agents that plausibly might give rise to the observed health outcome may begin.

Provided cases and controls are recruited in a population-based manner, and no geographic bias is introduced into the sampling frame, the tools presented in this paper may generate insights about geographic risk factors not considered in the initial design of the case-control study.

## Methods

In this section we first present a review of Q-statistics, and extend them to provide global, local and focused tests that account for risk factors and covariates. We next describe an experimental data set for bladder cancer in southeastern Michigan, and apply these new methods to this dataset to illustrate the approach.

### Q-statistics

Jacquez et al. [4] developed global, local and focused tests for case-control clustering of residential histories. Readers unfamiliar with Q-statistics may wish to refer to that original work. We now briefly present these techniques and then extend them to account for risk factors and covariates.

Define the coordinate **u**_*i*,*t *_= {*x*_*i*,*t*_, *y*_*i*,*t*_} to indicate the geographic location of the *i*^th ^case or control at time *t*. Residential histories can then be represented as the set of space-time locations:

**L**_*i *_= {**u**_*i*0_, **u**_*i*1_, ..., **u**_*iT*_} (Equation 2)

This defines individual *i *at location **u**_*i*0 _at the beginning of the study (time 0), and moving to location **u**_*i*1 _at time *t *= 1. At the end of the study individual *i *may be found at **u**_iT_. *T *is defined to be the number of unique location observations on all individuals in the study. Define a case-control identifier, *c*_*i*_, to be

$c_{i}=\{\begin{array}{ll} 1 & \text{if and only if }i\text{ is a case}\\ 0 & \text{otherwise} \end{array}\;\left(\text{Equation }3\right)$

Define n_a _to be the number of cases and n_b _to be the number of controls. The total number of individuals in the study is then *N *= *n*_a _+ *n*_b_. Let *k *indicate the number of nearest neighbours to consider when evaluating nearest neighbour relationships and define a nearest neighbour indicator to be:

$\eta_{i,j,k,t}=\{\begin{array}{ll} 1 & \text{if and only if }j\text{ is a }k\text{ nearest neighbor o}f\;i\text{ at time }t\\ 0 & \text{otherwise} \end{array}\;\left(\text{Equation }4\right)$

We define a binary matrix of *k*^th ^nearest neighbour relationships at a given time *t *as:

$\eta_{k,t}=\left[\begin{array}{ccccc} 0 & \eta_{1,2,k,t} & . & . & \eta_{1,N,k,t}\\ \eta_{2,1,k,t} & 0 & & & .\\ . & & . & & .\\ . & & & . & \eta_{N-1,N,k,t}\\ \eta_{N,1,k,t} & . & . & \eta_{N,N-1,k,t} & 0 \end{array}\right]\;\left(\text{Equation }5\right)$

This matrix enumerates the *k *nearest neighbours for each of the *N *individuals. The entries of this matrix are 1 (indicating that *j *is a *k *nearest neighbour of *i *at time *t*) or 0 (indicating *j *is not a *k *nearest neighbour of *i *at time *t*). It may be asymmetric about the 0 diagonal since nearest neighbour relationships are not necessarily reflexive. Since two individuals cannot occupy the same location, we assume at any time *t *that any individual has *k *unique *k*-nearest neighbours. The row sums thus are equal to *k *(η_*i*,•,*k*,*t *_= *k*) although the column sums vary depending on the spatial distribution of case control locations at time *t*. The sum of all the elements in the matrix is *Nk*. There exists a 1 × *T *+ 1 vector denoting those instants in time when the system is observed and the locations of the individuals are recorded. We can then consider the sequence of *T *nearest neighbour matrices defined by

$\eta_{k}^{T}$ = {η_*k*,*t *_∀ *t *= 0..*T*}     (Equation 6)

This defines the sequence of *k *nearest neighbour matrices for each unique temporal observation recorded in the data set, and quantifies how spatial proximity among the *N *individuals changes through time.

Alternative specifications of the proximity metric may be used – the metrics do not have to be nearest neighbour relationships in order for the Q-statistics to work. In this study we prefer to use nearest neighbour relationships because they are invariant under changing population densities, unlike geographic distance and adjacency measures. There also is some evidence that nearest neighbour metrics are more powerful than distance- and adjacency-based measures [28]. Still, one then may be faced with the question of "how many nearest neighbours (*k*) should I consider"? In certain instances one may have prior information that suggests that clusters of a certain size should be expected, and this can serve as a guide to specification of *k*. When prior information is lacking one may wish to explore several levels of *k*. In these instances Tango [29,30] advocates using the minimum p-value obtained under each level of *k *as the test statistic. In this paper we explore sensitivity by varying the number of nearest neighbors from *k *= 1,..10, 15, 25, 50 and 75. This allows us to evaluate how sensitive cluster location and strength is to the number of nearest neighbours. We then use concordance of results across different levels of *k *within the framework of strong inference to reach conclusions regarding clustering. Those concerned with strict statistical inference may wish to specify a single level of *k a priori *in order to avoid multiple testing, or to employ the min(p) approach of Tango.

Jacquez et al. (4) defined a spatially and temporally local case-control cluster statistic:

$Q_{i,k,t}=c_{i}\sum_{j=1}^{N}\eta_{i,j,k,t}\;c_{j}\;\left(\text{Equation }7\right)$

This is the count, at time *t*, of the number of *k *nearest neighbors of case *i *that are cases, and not controls. When *i *is a control *Q*_*i*,*k*,*t *_= 0.

To determine whether there is statistically significant case clustering of residential histories throughout the study area and when the entire study time period is considered (a spatially and temporally global test) we use:

$Q_{k}=\sum_{t=0}^{T}Q_{k,t}\;\left(\text{Equation }8\right)$

This is the sum, over all *T*+1 time points, of the temporally local and spatially global statistic $Q_{k,t}=\sum_{i=1}^{N}Q_{i,k,t}$. This will tell us whether there is global clustering of residential histories when all of the residential histories over the entire study period are considered simultaneously. Once global clustering is assessed, we next use Jacquez et al.'s *Q*_*i*,*k *_to identify local clusters of residential histories.

$Q_{i,k}=\sum_{t=0}^{T}Q_{i,k,t}\;\left(\text{Equation }9\right)$

For the *i*^th ^residential history, this is the sum, over all *T*+1 time points, of the local spatial cluster statistic *Q*_*i*,*k*,*t*_. It is the number of cases that are *k*-nearest neighbors of the *i*^th ^residential history (a case), summed over all *T*+1 time points. It will be large when cases tend to cluster around the *i*^th ^case through time. This statistic will be evaluated for each of the cases to identify those cases with low p-values. Notice the local statistics are a decomposition of the global statistic into local contributions, and the sum of the local statistics is equal to the global statistic.

We use the statistic *Q*_*F*,*k *_to determine whether bladder cancer cases cluster near the business addresses of industries known to emit bladder cancer carcinogens. This will allow us to evaluate whether there was statistically significant clustering about a given industry *F *(e.g. a specific metal-plating business) over the life of its operation. Suppose that one suspects that the cases may be clustering about a specific focus defined by the business address history:

**L**_*F *_= {**u**_*F*,0_, **u**_*F*,1_,.., **u**_*F*,*T*_} (Equation 10)

A test for spatial clustering of cases about the focus *F *at a given time *t *is then:

$Q_{F,k,t}=\sum_{j=1}^{N}\eta_{F,j,k,t}\;c_{j}\;\left(\text{Equation }11\right)$

Here η_*F*,*j*,*k*,*t *_is the nearest neighbor index indicating at time *t *whether the *j*^th ^individual is a *k*^th ^nearest neighbor of the geographic location of the focus defined by **u**_*F*,*t*_. The statistic *Q*_*F*,*k*,*t *_is the count of the number of *k*-nearest neighbors about the focus at time *t *that are cases. We use this statistic to evaluate clustering about the address histories of specific industries. We sum this statistic over all industries considered and the entire study period to obtain a global measure of focused clustering. We call this statistic $Q_{k}^{F}$ and use it to assess whether there is focused clustering when we consider all industries simultaneously.

We employ the duration-weighted versions of the above Q-statistics as presented in the Appendix to Jacquez et al. [4]. Jacquez et al. [4] also defined spatially and temporally local Q-statistics for individuals for evaluating those places of residence and intervals of time for which case clustering occurred. In this publication our focus is on the life course, and we leave further demonstration of the more ephemeral spatially and temporally local statistics for another paper.

### Randomization accounting for covariates and risk factors

In the absence of knowledge of other risk factors and covariates, simple randomization may be used when evaluating the statistical significance of the above statistics. This is accomplished by holding the location histories for the cases and controls constant, and by then sprinkling the case-control identifiers at random over the residential histories. This corresponds to a null hypothesis in which the probability of an individual being declared a case (*c*_i _= 1) is proportional to the number of cases in the data set, or:

$p(c_{i}=1|H_{0,I})=\frac{n_{1}}{n_{0}+n_{1}}\;\left(\text{Equation }12\right)$

Here *n*_1 _is the number of cases and *n*_0 _is the number of controls, and *H*_0,*I *_indicates a null hypothesis corresponding to Goovaerts and Jacquez's [14] type I neutral model of spatial independence. This null hypothesis assumes the risk of being declared a case is the same over all of the *N *case and controls.

#### Logistic model of the probability of being a case

In order to provide a more realistic null hypothesis we make the probability of being declared a case a function of the covariates and risk factors. Logistic models are used for binary response variables. Let **x **denote the vector of covariates and risk factors. Further, let p=Pr(*c *= 1|**x**) denote the response probability to be modeled, which is the probability of person *i *being a case. The linear logistic model is then:

logit(*p*) = log(*p*/1 - *p*) = α + **β'x **+ ε_*i *_    (Equation 13a)

and the equation for predicting the probability of being a case given the vector of covariates and risk factors for the *i*^th ^individual is:

$\hat{p}(c_{i}=1|x_{i})=\frac{e^{\alpha +\beta^{\prime }x_{i}}}{1+e^{\alpha +\beta^{\prime }x_{i}}}\;\left(\text{Equation }13\text{b}\right)$

Here the logit function is the natural log of the odds, α is the intercept parameter, and **β **is the vector of regression (slope) coefficients. One then fits the regression model to the vector of covariates and risk factors to calculate the intercept and slope parameters.

#### Randomization accounting for risk factors and covariates

We use approximate randomization to evaluate the probability of a given Q-statistic under the null hypothesis that the probability of being a case is a function of the covariates and risk factors specified in Equation 13b. To evaluate the reference distribution for a given Q-statistic we follow these steps.

Step 1. Calculate statistic (Q*) for the observed data. This may be any one of the global, local or focused Q-statistics calculated from the observations.

Step 2. Sprinkle the case-control identifier *c*_i _over the residential histories of the participants in a manner consistent with the desired null hypothesis, and conditioned on the observed number of cases. Assume we have *n*_a _cases, *N *participants and that P_i _is the probability of the *i*'th participant being a case. Notice the P_i _are provided by the logistic equation.

Step 2.1 Rescale the P_i _as follows: ${P^{\prime }}_{i}=P_{i}/\sum_{i=1}^{N}P_{i}$

Step 2.2 Map the ${P^{\prime }}_{i}$ to the interval 0 .. 1. For example, assume we have N = 2 participants, n_a _= 1 case and that P_1 _= .7 and P_2 _= .8. ${P^{\prime }}_{1}$ then maps to the interval [0 .. .7/1.5) and ${P^{\prime }}_{2}$ maps to the interval [0.7/1.5 .. 1.5/1.5).

Step 2.3 Allocate a case by drawing a uniform random number from the range [0..1). Set the case identifier equal to 1 (*c*_i _= 1) where *i *is the identifier corresponding to the study participant whose interval for ${P^{\prime }}_{i}$ contains the random number.

Step 2.4 Rescale as shown in Step 2.1 but not including the probability for the participant whose case identifier was assigned in step 2.3.

Step 2.5 Repeat Steps 2.2–2.4 until all of the n_a _case identifiers are assigned.

Step 2.6 Set the remaining *N *- *n*_a _case identifiers to 0, these are the controls.

Notice steps 2.1–2.6 result in 1 realization of the distribution of case-control identifiers.

Step 3. Calculate Q for the realization from Step 2.

Step 4. Repeat steps 2–3 a specific number of times (we used 999) accumulating the reference distribution of Q under the null hypothesis.

Step 5. Compare Q* to this reference distribution to evaluate the statistical probability of observing Q* given the known risk factors and covariates.

### Data

A population-based bladder cancer case-control study is underway in southeastern Michigan. Cases diagnosed in the years 2000–2004 are being recruited from the Michigan State Cancer Registry. Controls are being frequency matched to cases by age (± 5 years), race, and gender, and are being recruited using a random digit dialing procedure from an age-weighted list. At this stage of recruitment, controls are not adequately matched; therefore, age, race, and gender are included in the logistic regression model that accounts for covariates (below). To be eligible for inclusion in the study, participants must have lived in the eleven county study area for at least the past 5 years and had no prior history of cancer (with the exception of non-melanoma skin cancer). Participants are offered a modest financial incentive and research is approved by the University of Michigan IRB-Health Committee. The data analyzed here are from 219 cases and 437 controls (Table 2).

**Table 2:** Demographic and descriptive characteristics of 219 cases and 437 controls.

	Cases	Controls
**Age (yrs)**		
30–39	1%	2%
40–49	6%	8%
50–59	20%	9%
60–69	33%	48%
≥ 70	40%	32%
**Gender**		
Male	77%	87%
Female	23%	13%
**Race**		
Caucasian/White	95%	92%
African American/Black	1%	3%
Asian/Asian American	1%	2%
American Indian or Alaskan Native	3%	3%
**Education**		
≤ High School	39%	25%
Some Post-High School	30%	26%
College Graduate	19%	22%
Post-Graduate Education	12%	27%
		
Total Number of Residences	1624	3434
% of Person-Years in Study Area	66%	63%

Percentages do not always equal 100% due to rounding.

As part of the study, participants complete a written questionnaire describing their residential mobility. The duration of residence and exact street address were obtained, otherwise the closest cross streets were provided. Approximately 66% of cases' person-years and 63% of controls' person-years were spent in the study area. Of the residences within the study area, 88% were automatically geocoded or interactively geocoded with minor operator assistance. The unmatched addresses were manually geocoded using self-reports of cross streets with the assistance of internet mapping services (6%); if cross streets were not provided or could not be identified, residence was matched to town centroid (6%).

Address histories were collected for those industries believed to emit contaminants associated with bladder cancer. These were identified using the Toxics Release Inventory [26] and the Directory of Michigan Manufacturers Manufacturer Publishing Co., 1946, 1953, 1960, 1969, 1977, 1982). Standard Industrial Classification (SIC) codes were adopted, but prior to SIC coding, industrial classification titles were selected. Characteristics of 268 industries, including, but not limited to, fabric finishing, wood preserving, pulp mills, industrial organic chemical manufacturing, and paint, rubber, and leather manufacturing, were compiled into a database (Table 3). Each industry was assigned a start year and end year, based on best available data. Industries were geocoded following the same matching procedure as for residences: 89% matched to the address, 5% were placed on the road using best informed guess, and as a last resort, 6% were matched to town centroid.

**Table 3:** SIC codes for industries considered to plausibly be associated with bladder cancer.

Standard Industrial Classification Code	Description of Industry
211_	Cigarettes
212_	Cigars
213_	Tobacco
214_	Tobacco
223_	Wool, Woven Fabric
226_	Cotton Fabric Finishers
2491	Wood Preserving
2611	Pulp Mills
2621	Paper Mills
2631	Paperboard Mills
2816	Inorganic Pigments
2819	Chemicals, Industrial Inorganic
2821	Plastics, Synthetic Resins, Elastomers
2822	Synthetic Rubber
2844	Perfumes, Cosmetics
2851	Paint, Varnish, Lacquer, Enamel
2865	Cyclic Crudes, Dyes, Organic Pigments
2869	Chemicals, Industrial Organic
287_	Fertilizers, Pesticides
2893	Printing Ink
2895	Carbon Black
301_	Tires and Tubes
302_	Rubber, Plastic Footwear
303_	Rubber, Reclaimed
304_	Rubber, Plastic Hose and Belting
306_	Rubber Products Fabricated
311_	Leather Tanning and Finishing
313_	Boot, Shoe Cut Stock and Findings
314_	Footwear
315_	Gloves, Mittens, Leather
316_	Luggage, Leather
317_	Leather Goods, Personal
319_	Leather Goods, Misc.
3312	Blast Furnaces, Steel and Rolling Mills
333_	Smelting
334_	Secondary Smelting
3691	Batteries, Storage
3692	Batteries, Wet and Dry

### Statistical analyses

The Q-statistic for examining space-time clustering was computed using TerraSeer's STIS software [31]. To account for covariates and risk factors in the Q-statistic, we conducted unconditional logistic regression analysis using "proc logistic" in Statistical Analysis System^® ^(version 8.0; SAS Institute, Inc., Cary, NC). The following covariates and risk factors have been summarized by Silverman et al [25] as being significant for bladder cancer and were included in the logistic regression model. The variables used in the model were defined as follows.

**Age**: Participant's age at time of interview

**Gender**: 1 = Male, 2 = Female

**Education**: 1 = <8 years, 2 = 8–11 years, 3 = 12 years or high school graduate, 4 = post high school training, 5 = some college, 6 = college graduate, 7 = postgraduate education

**Race**: 1 = white, 2 = black, 3 = other

**Number of Cigarettes Smoked**: 0 = never smoked, 1 = smoked < 10 cigarettes daily, 2 = smoked 11–20 cigarettes daily, 3 = smoked 21–30 cigarettes daily, 4 = smoked > 30 cigarettes daily

The parameter estimates of the model were used to estimate a probability of being a case for each participant and included in the covariate-adjusted analysis of the Q-statistic in the STIS software.

## Abbreviations

GIS: Geographic Information System

IRB: Institutional Review Board

SIC: Standard Industrial Classification

STIS: Space Time Intelligence System

TRI: Toxics Release Inventory

## Competing interests

The authors are affiliated with organizations (The University of Michigan, BioMedware) that receive grant funding to conduct the research described in this publication. BioMedware developed the STIS software used in this research.

## Authors' contributions

GMJ developed the Q-statistics, conducted the analyses and drafted the manuscript. JRM and GMJ collaborated on the design of the analyses and manuscript revisions. GAA geocoded the residential histories. PG developed the geostatistical techniques. AK coded the Q-statistics in the Space-Time Intelligence System software. MW advised on the design of the bladder cancer study. JN directed the bladder cancer study and provided the residential histories data.

## Supplementary Material

Additional File 1 Animation of local clustering of cases at *k *= 7 accounting for smoking, gender, age, race and education. Controls indicated with a "+", cases by circles. Blue is a not significant local Q-statistic for that case, yellow p < 0.05, orange p < 0.01, Red p < 0.001. Click here for file

Additional File 2 Animation of focused clustering about business address histories at k = 8. Industries are indicated by triangles, controls by a "+", cases by circles. Triangles are color-coded based on statistical significance after covariate adjustment. Green is not significant, yellow p < 0.05, orange p < 0.01, Red p < 0.001. Click here for file
